# Using a metaphor of baskets and copper pots to identify “what work, whose work” in truth, rights, responsibilities, and reconciliation in public health

**DOI:** 10.17269/s41997-025-01116-3

**Published:** 2025-10-17

**Authors:** Danièle Behn Smith, Jessica Chenery, Naomi Dove, Kate Jongbloed

**Affiliations:** 1 BC Office of the Provincial Health Officer, Victoria, BC Canada; 2 Chee Mamuk, Vancouver, BC Canada; 3 BC Centre for Disease Control, Vancouver, BC Canada; 4 University of Victoria, Victoria, BC Canada

**Keywords:** Settler colonialism, White supremacy, Indigenous-specific racism, Reconciliation, Public health, Colonialisme de peuplement, Suprématie blanche, Racisme particulier à l’encontre des Autochtones, Réconciliation, Santé publique

## Abstract

Ten years since the Truth & Reconciliation Commission Report, Canadian institutions—including public health systems—have yet to advance the Calls to Action in a sustained, transformative way. As public health leaders in the territory now known as British Columbia, we witness tension as colleagues grapple with, “What is the work of Truth & Reconciliation? Whose work is it?”. Too often, truth and reconciliation is delegated to a small Indigenous team (or, individual) dangling, isolated off the side of an organizational chart. We offer a metaphor highlighting two interconnected, but distinct areas of work to advance truth and reconciliation in public health. One is the work of reclaiming and resurgence of languages, culture, medicines, and connection to territory, undertaken by and for First Nations, Inuit, and Métis Peoples. The other is eradicating Indigenous-specific racism and white supremacy to advance cultural safety. It is not up to Indigenous people to eradicate racism; as it is constructed, maintained, and perpetuated by settlers, settlers are those with the power to eradicate it. As we move towards the anniversary of the TRC, we share a metaphor that helps our settler colleagues understand and claim their responsibility in truth, rights, and reconciliation in public health.

## Résumé

Presque 10 ans après le rapport de la Commission de vérité et réconciliation (CVR), les institutions canadiennes, y compris les systèmes de santé publique, n’ont toujours pas répondu aux Appels à l’action de façon soutenue et transformative. En tant que responsables de la santé publique sur le territoire aujourd’hui appelé la Colombie-Britannique, nous sommes témoins de tensions lorsque nos collègues essaient de comprendre ce qu’est le travail de Vérité et de Réconciliation et qui en est chargé. Trop souvent, la Vérité et la Réconciliation sont déléguées à une petite équipe (ou à une seule personne) autochtone laissée en suspens, isolée sur le côté de l’organigramme. Nous proposons la métaphore de deux domaines d’activité interconnectés, mais distincts pour faire progresser la Vérité et la Réconciliation en santé publique. Le premier est le travail de réclamation et de résurgence des langues, des cultures, des remèdes et du rapport au territoire, entrepris par et pour les Premières Nations, les Inuits et les Métis. Le second est l’éradication du racisme particulier à l’encontre des Autochtones et de la suprématie blanche pour favoriser la sécurisation culturelle. Ce n’est pas aux peuples autochtones d’éradiquer le racisme; comme il est construit, maintenu et perpétué par les colonisateurs, ce sont les colonisateurs qui ont le pouvoir de l’éradiquer. À l’approche de l’anniversaire de la CVR, nous espérons que cette métaphore aidera nos collègues colonisateurs à comprendre la Vérité, les Droits et la Réconciliation en santé publique et à revendiquer leur responsabilité à cet égard.

**Graphical Abstract:**

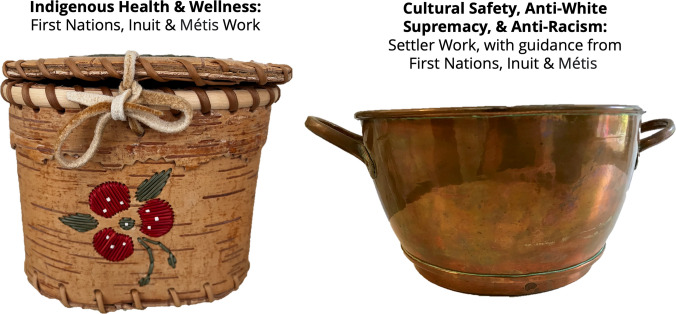

## Introducing ourselves

We are two “duos” or pairs of Indigenous and non-Indigenous public health leaders working together in two provincial public health organizations—the BC Office of the Provincial Health Officer and the BC Centre for Disease Control (BCCDC). The overarching purpose of our collective work is to advance the rights, health, and wellness of Indigenous Peoples living in BC. Danièle is Métis from the Red River Valley through her mother and Eh Cho Dene from Fort Nelson First Nation through her father. She works as BC Deputy Provincial Health Officer—Indigenous Health and champions efforts to unlearn and undo systemic white supremacy and Indigenous-specific racism to advance inherent Indigenous rights. Jessica is Coast Salish on Vancouver Island (Shíshálh and Penelakut First Nations), Portuguese, and Scottish. Jessica enjoys her work as the Director of Chee Mamuk (“New Work” in Chinook jargon), a self-determining Indigenous-led program within the BCCDC. Jessica leads with love and is a strong believer in the power of bringing community members together to engage in meaningful conversations and have fun, influencing the system from the ground up to better serve Indigenous people. Naomi is a white settler of British and Newfoundland descent raised on the territory of the Anishinaabe and Métis People of Treaty 3 and Treaty 3 Adhesion in rural Northwestern Ontario. Naomi is honoured to be invited in as a guest to work with the Chee Mamuk team as Medical Director in a duo with Jessica. Kate is a white occupier and epidemiologist whose work focuses on daylighting and addressing the impacts of white supremacy, settler colonialism, and Indigenous-specific racism on health and wellbeing. She is a BCCDC Senior Scientist, University of Victoria adjunct professor, and the other half of a duo with Danièle.

In her book, *True Reconciliation*, Puglaas Jody Wilson-Raybould shares, “Social and economic disparities and Indigenous rights are completely intertwined and interconnected. They are indivisible. We will not and cannot address social and economic disparities without addressing the recognition and implementation of Indigenous rights. They are two tracks that criss-cross in countless ways and do not run parallel and separate from each other. We must move down both tracks in a conscious, coherent, consistent way if we want to effect true reconciliation.”(Wilson-Raybould, 2022) Teachings from Danièle’s dad, Pehcheh Behn (son of Mary and George Behn), remind us that rights and responsibilities go hand in hand, reflecting the responsibilities that First Nations people have to the lands and waters since time immemorial. As a result, we use the terms truth, rights, responsibilities, and reconciliation to label our area of work.

## Truth, rights, responsibilities, and reconciliation in public health

When the Truth & Reconciliation Commission’s Final Report was shared in 2015, late Commissioner Justice Mizhana Gheezhik Murray Sinclair (Peguis First Nation) said, “We have described for you a mountain, we have shown you the path to the top. We call upon you to do the climbing.” Thousands of survivors of residential schools and their descendants took Canada to court; their testimony resulted in 94 Calls to Action, which became the responsibility of all Canadians. Since that time, we have received many more clear and detailed instructions from First Nations, Inuit, and Métis People representing solutions to health disparities. These foundational obligations include 231 Missing & Murdered Indigenous Women, Girls, & 2SLGBTQQIA+ Calls for Justice (National Inquiry into Missing and Murdered Indigenous Women Girls, 2019) and 46 articles of the UN Declaration on the Rights of Indigenous Peoples (UN General Assembly, 2007) which is now law in Canada (*United Nations Declaration on the Rights of Indigenous Peoples Act (S.C. 2021,* *c.14)*, 2021). Yet, when Canadian institutions are evaluated against these commitments, we are found lacking (Jewell & Mosby, 2023).

Evidence of the presence and persistence of Indigenous-specific racism in every nook and cranny of Canadian institutions, including public health, continues to grow (Council of the Atikamekw of Manawan & Council de la Nation Atikamekw, 2020; Sharma et al., 2023; Turpel-Lafond, 2020; Wabano Centre for Aboriginal Health in Partnership with the Ottawa Aboriginal Coalition, 2022). Racism as a public health crisis affecting the health of individuals and populations is recognized in statements from the Canadian Public Health Association (Canadian Public Health Association, 2018); Lancet (Devakumar et al., 2020); and Toronto’s Board of Health (Toronto Board of Health, 2020), to name a few. Racism as a public health crisis reinforces and exacerbates pressing public health issues, including climate change (Jones, 2019), toxic drug crisis (FNHA.ca, 2024), and COVID-19 (Chief Public Health Officer of Canada, 2020, 2021). Racism is deeply embedded within public health structures and practices (Canadian Public Health Association, 2018). Examples of Indigenous-specific racism in public health in Canada past and present include denial of smallpox treatment (Ostroff, 2017); nutritional experimentation in residential schools (Mosby, 2013); segregated tuberculosis sanitoria and Indian Hospitals (Lux, 2016); construction of Indigeneity as “risk” (Mikkonen et al., 2020); concentration of decision-making power and authority within institutions that have systematically excluded Indigenous people and leaders (Came & Humphries, 2014); inequitable access to public health benefits (First Nations Health Authority & Office of the Provincial Health Officer, 2024); inadequate Indigenous data governance (Verhaeghe et al., 2021); imposition of public health legislation without regard for Indigenous law (Verhaeghe et al., 2021); and paternalism.

In *Visioning the Future: First Nations, Inuit, & Métis Population and Public Health*, Indigenous public health leaders from across Canada emphasize the importance of establishing population and public health care systems free of racism (National Collaborating Centre for Indigenous Health, 2021). Public health systems have clear obligations and mandates to uphold Indigenous rights and address Indigenous-specific racism towards meaningful and lasting reconciliation. The 2021 Chief Public Health Officer of Canada’s report states that, “we are now at a pivotal moment” with the opportunity to actively “decide what kind of public health system, and ultimately, society we want to have, moving forward” (Chief Public Health Officer of Canada, 2021). Public health thinkers propose a “Fifth Wave” that breaks from mechanistic, paternalistic approaches, embracing complexity, interdependence, and cooperation to nurture human life, spirit, and self-healing (Hanlon et al., 2011) A key element in applying a Fifth Wave approach requires public health systems to embed Indigenous ways of knowing and being.

## What work, whose work?

We learned from First Nations colleagues working in public health that often, they are asked to lead all work connected to “Indigenous Health” in their organizations. This label blinds the root problems of racism and white supremacy, and where responsibility to respond lies. Foundational obligations that all Canadians have to Indigenous Peoples outline clear instructions for public health leaders, practitioners, and researchers. Yet, settler colleagues often remove themselves from responsibility, ranging from well-meaning but problematic deference to Indigenous Peoples to outright denial and resistance (Carleton, 2021).

In asking, “what work” and “whose work,” we understand there are two distinct but interconnected types of work in truth, rights, and reconciliation. We use the metaphor of baskets and copper pots to help Indigenous and non-Indigenous colleagues alike understand and communicate their roles in truth, rights, and reconciliation.

## Basket work

Basket work is health and wellness work that promotes cultural continuity. This work is done by and for First Nations, Inuit, and Métis Peoples and calls upon the wisdom of their unique and distinct knowledges. This basket of work has always been here, and is still here, though it is deliberately and systematically undermined by imposed settler colonial systems. We represent this with an image of a birch basket from the Dene people of Treaty 8 territory, made by birch bark basket artists from Liard River First Nation. Danièle gifted this basket to her mom, who resides on Treaty 1 territories and homeland of the Métis.

## Copper pot work

Copper pot work is the work of settlers, including settler leaders, in the mainstream health system. It is the work of arresting the systems of oppression that settlers have put in place—white supremacy, racism, settler colonialism—so that we can begin to fully uphold Indigenous rights. Copper pot work must be undertaken with guidance from First Nations, Inuit, and Métis Peoples, as settlers have not yet demonstrated trustworthiness. The copper pot—made in Kate’s great Opa’s machine shop in Holland—is a reminder that we are newcomers, carrying a different set of understandings and perspectives than the First Peoples of these territories. Looking closely, you can see Kate’s reflection, a reminder to settler colleagues that we are in the work and have responsibility to mobilize white and settler privilege to advance truth and reconciliation.

### Anishinaabe Elder’s Teaching About Copper

*Once when we were sharing this metaphor, Anishinaabe Elder Phyllis Williams from Curve Lake First Nation generously offered teachings about sacred copper vessels being used in ceremony because of the cleansing and purifying powers of copper. We now see an additional element to the metaphor which is that the hard work that settlers must do is sacred.*

## Distinct roles and responsibilities in relation to “Indigenous health”

Basket work and copper pot work are often conflated and incorrectly labelled as “Indigenous health.” By centering “Indigenous” as both the problem and the solution, this mislabeling blinds settlers from the root causes of Indigenous peoples’ poor health and from their critical roles in anti-racism and anti-white supremacy. By naming these two different areas of work, our distinct roles and responsibilities as Indigenous and non-Indigenous peoples to promote the health of Indigenous peoples become clear.

Basket and copper pot work are equally important; both must be fully funded and resourced. Most importantly, these distinct areas of work need to be undertaken by the right people. Indigenous Peoples are too often called upon to do the “copper pot” work of settlers. It is tantamount to asking an Indigenous colleague, “have you fixed our racism yet?” This can be harmful, and it diverts Indigenous colleagues’ time, energy, and resources away from important “basket work” of maintaining cultural continuity. Settlers must dismantle the Indigenous-specific racism, white supremacy, and settler colonialism impeding basket work from flourishing fulsomely and wholistically throughout public health systems, with guidance from First Nations, Inuit, and Métis Peoples.

## No one should do this work alone

One of our guiding principles is that no one should do this work alone; our Basket-Copper Pot Duos demonstrate this powerful way to advance the work. First, it supports our wellness. Second, we can call upon the multi-dimensional skills and perspectives across each duo: two-eyed seeing in action (Marshall et al., 2015). Third, it is an effective strategy to mitigate the tyranny of the majority in spaces where there still is only one Indigenous person at the table (Came & Humphries, 2014). Now, we are exploring opportunities to be deliberate about basket-copper pot duo hiring in our spheres of influence.

## Conclusion

Residential school survivors and their descendants did the heavy lifting of sharing their testimony to provide Canadians with the 94 TRC Calls to Action. Ten years since their release, Indigenous people have largely shouldered the burden of advancing truth, rights, and reconciliation work, including in public health. We offer our basket and copper pot metaphor so that our settler colleagues might step further into their responsibility to truth, rights, and reconciliation so that 10 years from now, we have a different story to tell.

## Data Availability

Not applicable.
